# Genetics of High-Grade Endometrioid Adenocarcinoma of the Ovary With Yolk Sac Differentiation: A Case Report

**DOI:** 10.7759/cureus.98594

**Published:** 2025-12-06

**Authors:** Lindsey Ammann, Yelena Piazza, Hatem Kaseb

**Affiliations:** 1 School of Medicine, University of Central Florida College of Medicine, Orlando, USA; 2 Clinical Sciences, University of Central Florida College of Medicine, Orlando, USA

**Keywords:** divergent differentiation, high-grade endometroid adenocarcinoma, ovarian epithelial tumors, perimenopausal woman, yolk sac differentiation

## Abstract

Yolk sac tumors (YST) are relatively rare ovarian neoplasms that typically occur in young women, whereas yolk sac differentiation in ovarian epithelial tumors represents an exceedingly rare malignancy of perimenopausal and postmenopausal women. The latter is usually associated with Müllerian epithelial neoplasms and is somatically derived. Accurate identification of tumor lineage is essential for proper classification and treatment, especially in mixed tumors with overlapping morphological and immunohistochemical features and shared mutations. Most of the reported cases have lacked genetic testing. Here, we present a case of high-grade endometrioid adenocarcinoma with yolk sac tumor differentiation in a perimenopausal woman. The tumor demonstrated an unusual morphology and immunohistochemical profile, raising the possibility of either a common early precursor or divergent differentiation. Genomic analysis revealed pathogenic mutations in *CTNNB1*, *ARID1A*, and *PIK3CA*, supporting an endometrioid origin. This case highlights the diagnostic challenges posed by rare ovarian tumors and emphasizes the role of molecular profiling in clarifying tumor phenotype. Pathologists should be aware of the potential association between epithelial tumors and yolk sac tumor differentiation, particularly in older patients, to avoid misdiagnosis and to ensure appropriate therapeutic strategies.

## Introduction

Ovarian pure yolk sac tumors (YST) are the second most common germ cell pediatric neoplasms with well-known sensitivity to chemotherapy [1,2]. Ovarian adenocarcinoma with YST differentiation is an extremely rare entity [3]. Among women who are perimenopausal/postmenopausal, YSTs typically present in association with other epithelial tumors (endometrioid adenocarcinoma, serous carcinoma, clear cell carcinoma, or carcinosarcoma) and are characterized by variable and diverse histological patterns [4]. Here, we present a case of endometroid adenocarcinoma with yolk sac differentiation that occurred in a perimenopausal woman with unusual morphological, immunohistochemical, and genetic profiles. Pure YSTs can be challenging to diagnose due to the morphological variability owing to their primitive endodermal nature [5]. Ovarian carcinomas with yolk sac differentiation are rare, and understanding their pathogenesis is essential for optimization of clinical management [6,7].

## Case presentation

A 49-year-old multiparous woman (G3P3) with a history of bilateral tubal ligation and multiple caesarean sections presented with a progressively enlarging pelvic mass and worsening pelvic pain over eight months. The patient’s mother had a history of an unknown gynaecological cancer. Abdominal examination demonstrated significant bilateral lower abdominal tenderness with pain and fullness palpated up to the supraumbilical region. Pelvic MRI without contrast revealed a 15 cm complex cystic left ovarian mass extending from the pelvis to the mid abdomen, with an inferior 5.5 cm solid component. The right ovary showed several cystic follicles and no evidence of malignancy. No lymphadenopathy was identified.

Laboratory analysis revealed elevated serum levels of alpha-fetoprotein (AFP), cancer antigen 125 (CA-125), cancer antigen 19-9 (CA 19-9), and carcinoembryonic antigen (CEA) (Table 1).

**Table 1:** Summarized patient’s tumor marker levels.

Analyte (Full Name)	Abbreviation	Result	Reference Range	Unit of Measurement
Alpha-fetoprotein	AFP	3,000	< 8	ng/mL
Cancer antigen 125	CA-125	378	< 30.2	U/mL
Cancer antigen 19-9	CA 19-9	235	< 30.2	U/mL
Carcinoembryonic antigen	CEA	20.3	< 5.0	ng/mL

**Figure 1:**
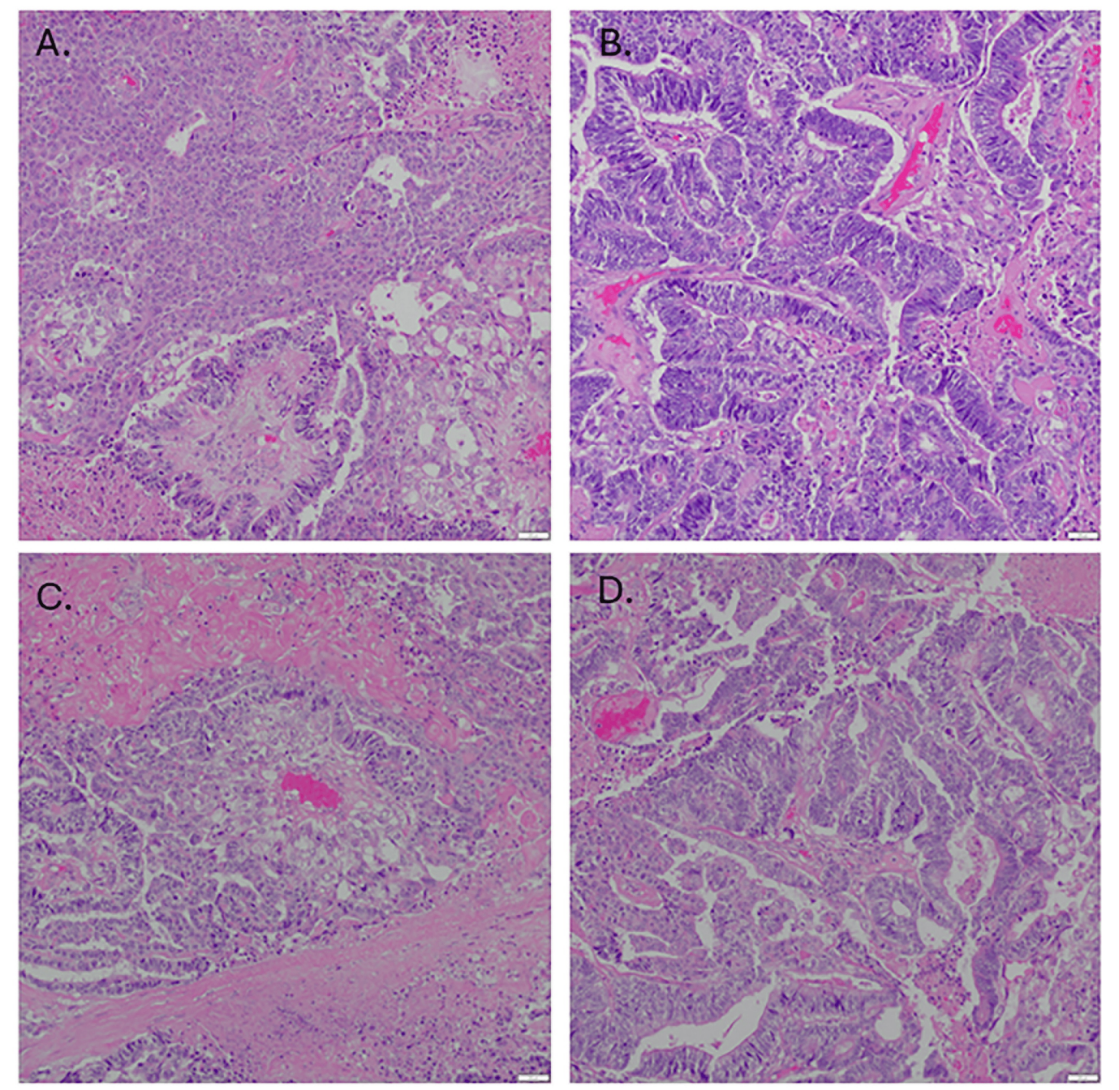
Morphological assessment of the tumor and omental implant. A. Solid and papillary pattern with squamoid, columnar, and clear cells (H&E, x40). B. Glandular and trabecular pattern with columnar cells (H&E, x100). C. Endodermal pattern with Schiller-Duval body (H&E, x200). D. Omental implant with multiple patterns (H&E, x100), necrotic changes and hemorrhage are seen (A-D).

The patient subsequently underwent a hysterectomy with bilateral salpingo-oophorectomy, resection of regional lymph nodes, and staging (including pelvic washings and omental biopsies). Grossly, the left ovary was replaced by a multi-cystic mass with tan-brown solid areas and regions of hemorrhage and necrosis. The ovarian capsule was involved by a neoplasm. The omentum demonstrated multiple foci of tumor involvement. Microscopic examination revealed multiple morphological patterns that included variable glandular patterns, solid pattern, some squamoid-like foci with abundant cytoplasm, Schiller-Duval bodies with clear cell appearance, as well as primitive appearing cells. The tumor cells showed moderate to severe nuclear atypia, multiple mitosis, and extensive areas of necrosis and hemorrhage. On the periphery of the ovary, there was an area of endometrioid adenofibroma with squamous metaplasia (Figure 1).

**Figure 2:**
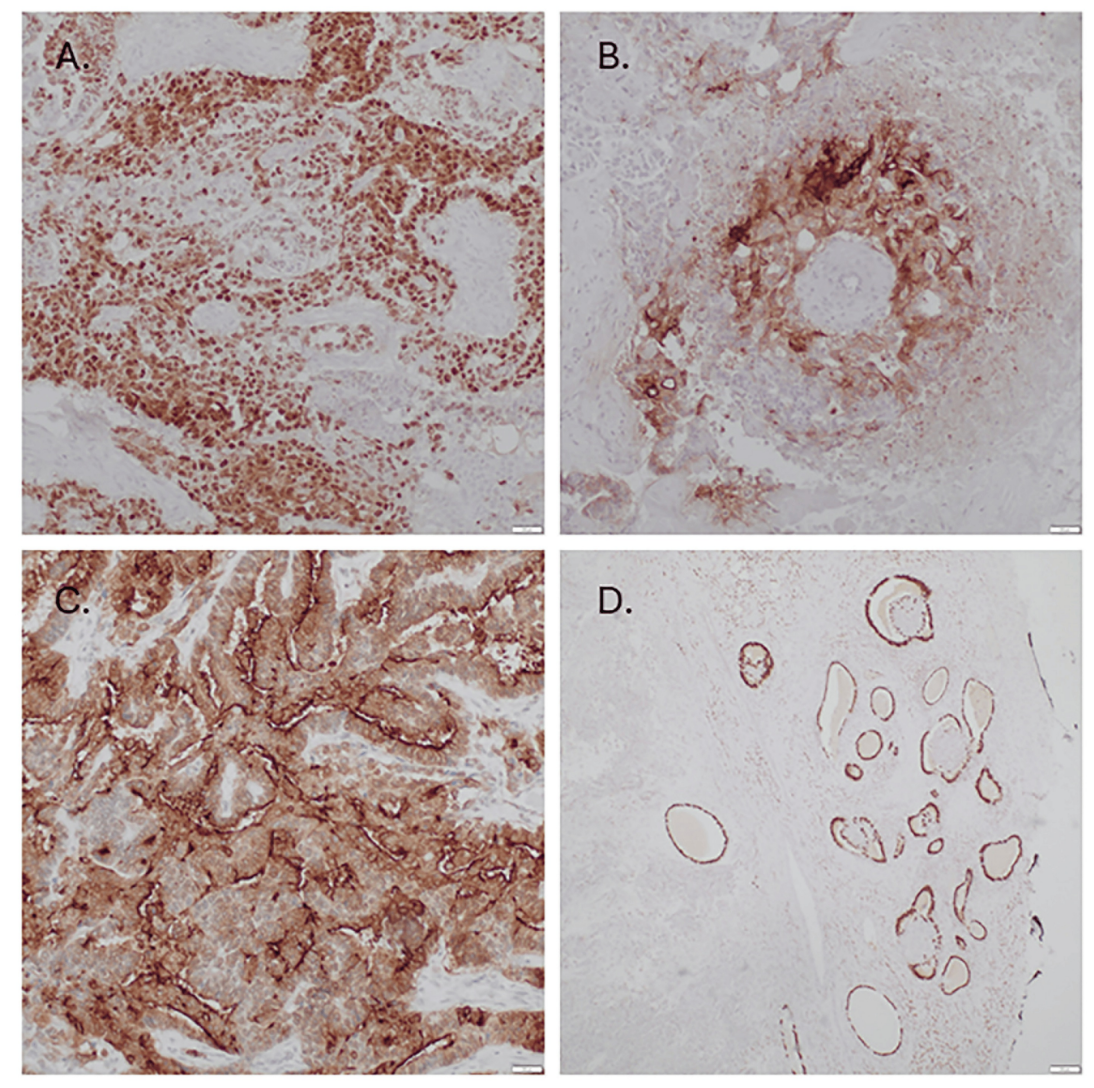
Immunohistochemical of the tumor and endometrial adenofibroma. A. Strong SALL-4 reactivity of neoplastic cells (x100). B. Focal reactivity with AFP seen in the cells with clear cytoplasm (x200). C. Tumor cells demonstrate strong reactivity with glypican-3 (x100). D. Cells of adenofibroma demonstrate nuclear ER-reactivity (x40).

Immunohistochemical studies were performed on the formalin-fixed paraffin-embedded sections. All tumor cells were of epithelial origin and were reactive with AE1-AE3 and focally positive with PAX-8, predominantly in the cystadenoma. Tumor cells exhibited a yolk sac tumor profile that included strong reactivity with SALL-4 and focal/weak reactivity with AFP and glypican-3 (Figure 2).

The tumor cells were negative for vimentin, p16, ER, and PR receptors. A few cells were positive for GATA3. Immunohistochemical panels for clear cell carcinoma, serous carcinoma, embryonal carcinoma, and choriocarcinoma were negative. Mismatch repair protein testing by immunohistochemistry (MLH1, PMS2, MSH1, and MSH2) showed intact expression, ruling out Lynch syndrome. Microscopic examination of four regional lymph nodes was negative for neoplasia; multiple peritoneal implants, mesenteric, and omental implants showed metastases of tumor with yolk-sac differentiation. Solid next-generation exome sequencing (Gatorseq NGS Panel; 718 genes; University of Florida) utilizing Illumina NovaSeq showed few pathogenic mutations in *CTTNB, ARID1A, *and* PIK3CA* (Table 2).

**Table 2:** Results of the exome NGS panel performed on the case.

Tier 1 (pathogenic variants)	Tier II (likely pathogenic variants)	Tier III (variants of undetermined significance)
CTNNB1	ARID1A	AXL
MUTYH	PIK3R1	BCOR
PIK3CA	SPOP	CBLC
		FANCE
		FGF23
		FLT3
		GABRA6
		KDM5C
		LRP1B
		MGA
		MSH3
		MSH3
		PBRM1
		PIK3C2G
		POLD1
		PPP2R1A
		RIPK1
		RPS6KA4
		SH2B3
		SLX4

The patient was clinically staged as stage IIIC ovarian high-grade endometrioid carcinoma with yolk-sac differentiation. The patient received three cycles of bleomycin, etoposide, and cisplatin (BEP), a germ cell tumor protocol with a favorable response. She tolerated the therapy, apart from developing anemia and nausea. AFP levels went down to 132 ng/ml, and the tumor burden was decreased.

## Discussion

YST typically arises in premenopausal women and is rare in postmenopausal women [3]. While most YSTs originate from germ cells, rare cases can arise from somatic tumor cells, particularly epithelial carcinomas [7]. In the current case, a perimenopausal woman presented with endometrioid adenocarcinoma of the ovary with YST differentiation. These tumors often present diagnostic challenges due to the overlapping morphologic features with high-grade serous carcinomas and other germ cell tumors [6].

A case series evaluating ovarian tumors with YST differentiation found that only one out of seventeen tumors was an endometrioid carcinoma with YST differentiation, underscoring the rarity of this tumor [2]. Immunohistochemistry helps in distinguishing such tumors, especially those that are mixed. The patient’s tumor lacked WT1, p16, and p53 overexpression, which helped exclude high-grade serous carcinoma [8]. A diagnosis of high-grade endometrioid adenocarcinoma with YST differentiation was rendered based on the morphologic features and immunopositivity for SALL-4, glypican-3, and AFP (Figure 2). These three immunohistochemical markers are highly sensitive for YST; however, not entirely specific [8]. SALL-4 is a germ cell marker that is typically positive in dysgerminoma and is usually used to differentiate germ cell tumors from epithelial neoplasms [8]. Tumor cell positivity for glypican-3 and AFP supported yolk sac differentiation [9]. Glypican-3 is considered highly specific for YST [6]. The tumor morphology and negative α-inhibin helped exclude sex cord stromal tumor [6]. GATA3 is a useful marker in highlighting the reticular-microcystic patterns of primary YST but not the glandular, hepatoid, or solid patterns [5]. In our case, GATA3 staining was focal and is consistent with prior observations [5]. CK7 and EMA are traditionally negative in pure YSTs [10]. The staining of the tumor with CK7 and EMA, in addition to the YST markers highlighted previously, thus further supports the diagnosis of endometrioid adenocarcinoma with YST differentiation [8].

The explanation of the variability of morphology and immunohistochemical stains could be that this tumor either originated from a common early precursor or through divergent/aberrant differentiation [8,11]. Aberrant differentiation is a phenomenon in which a new cell phenotype arises through a process of transformation of an original somatic precursor neoplasm [2,7,10]. While many ovarian divergent differentiation cases demonstrate distinct germ cell and epithelial carcinoma areas [2,10], there is a possibility that the germ cell component transitioned from and obscured the epithelial component, as evident in the current case [2]. 

Of particular interest in this case is the tumor’s genetic profile: the identification of pathogenic/likely pathogenic mutations in *CTNNB1 *(β-catenin), *ARID1A*, and *PIK3CA* strongly supports a diagnosis of endometrioid adenocarcinoma with somatic YST differentiation rather than a primary germ cell yolk sac tumor because *CTNNB1* and *PIK3CA* mutations are endometrioid carcinoma-specific mutations [11,12]. These mutations are frequently observed in endometrioid ovarian carcinomas and are associated with tumors arising from endometriosis [13]. In contrast, pure ovarian YST of germ cell origin typically lack these mutations with some cases may show *KRAS, ARID1A, KIT* mutations; most cases often harbor distinct germ cell-related alterations, such as chromosomal 12p abnormalities [13,14]. Recent molecular analyses have demonstrated that in mixed tumors, the YST and carcinoma components can harbor shared somatic mutations, including *CTNNB1 *and* ARID1A*, implying a common clonal origin and supporting the theory of somatic derivation [11,15]. In the current case, the concurrent presence of *CTNNB1, ARID1A, *and* PIK3CA* mutations, which are well-described in endometrioid carcinomas, as well as the absence of germ cell tumor-specific genetic alterations, provides strong molecular evidence that the YST phenotype observed was somatically derived. This genetic profile reinforces the classification of the tumor in this case as an endometrioid adenocarcinoma with YST differentiation and emphasizes the value of molecular profiling in distinguishing between primary germ cell tumors and somatic carcinomas with germ cell-like features [13,15].

## Conclusions

Ovarian tumors that undergo germ cell differentiation may be less responsive to chemotherapy directed solely at germ cell tumors. In these cases, platinum-based regimens designed to treat both epithelial ovarian neoplasms and germ cell tumors may offer better clinical outcomes. In conclusion, this case highlights the complex diagnostic challenges that pathologists may face when diagnosing ovarian tumors. YSTs are generally hard to diagnose due to the morphological and immunohistochemical overlap with other tumors. Another challenge in diagnosis is to exclude mixed tumors, which typically requires careful examination and adequate sampling. Genetic profiling can confirm somatic derivation and guide appropriate multimodal therapy.

## References

[1] Faure Conter C, Xia C, Gershenson D (2018). Ovarian yolk sac tumors: does age matter?. Int J Gynecol Cancer.

[2] McNamee T, Damato S, McCluggage WG (2016). Yolk sac tumours of the female genital tract in older adults derive commonly from somatic epithelial neoplasms: somatically derived yolk sac tumours. Histopathology.

[3] Sookram J, Levin B, Barroeta J, Kenley K, Mehta P, Krill LS (2019). A case of ovarian endometrioid adenocarcinoma with yolk sac differentiation and Lynch syndrome. Gynecol Oncol Rep.

[4] Euscher ED (2017). Unusual presentations of gynecologic tumors: extragonadal yolk sac tumor of the vulva. Arch Pathol Lab Med.

[5] Schuldt M, Rubio A, Preda O, Nogales FF (2016). GATA binding protein 3 expression is present in primitive patterns of yolk sac tumours but is not expressed by differentiated variants. Histopathology.

[6] Xing F, Jiang L, Wang T, Li W, Yang P, Qu G, Bao L (2020). High-grade serous carcinoma of fallopian tube with yolk sac tumor differentiation in a postmenopausal patient. Int J Clin Exp Pathol.

[7] Kane SV, Wuntkal R, Ramadwar M, Tongaonkar HB (2004). Ovarian endometrioid carcinoma with yolk sac component in a young patient: a diagnostic and therapeutic dilemma. Aust N Z J Obstet Gynaecol.

[8] McCarthy WA, Masand RP (2016). Ovarian yolk sac tumor with high-grade serous carcinoma in a 62-year-old woman. Int J Surg Pathol.

[9] Mills AM, Jenkins TM, Dibbern ME, Atkins KA, Ring KL (2024). Yolk sac differentiation in endometrial carcinoma: incidence and clinicopathologic features of somatically derived yolk sac tumors versus carcinomas with nonspecific immunoexpression of yolk sac markers. Am J Surg Pathol.

[10] Ohishi Y, Kaku T, Kaneki E, Wake N, Tsuneyoshi M (2007). Malignant ovarian tumor composed of endometrioid adenocarcinoma, clear cell adenocarcinoma, squamous cell carcinoma, yolk sac tumor and immature teratoma with prominent neuroectodermal and rhabdomyosarcomatous differentiation: a case study. Gynecol Oncol.

[11] Halling GC, Udager AM, Skala SL (2023). Endometrial, ovarian, and peritoneal involvement by endometrioid carcinoma, yolk sac tumor, and endometriosis: molecular evidence for a shared precursor. Int J Gynecol Pathol.

[12] Acosta AM, Sholl LM, Cin PD, Howitt BE, Otis CN, Nucci MR (2020). Malignant tumours of the uterus and ovaries with Mullerian and germ cell or trophoblastic components have a somatic origin and are characterised by genomic instability. Histopathology.

[13] Skala SL, Liu CJ, Udager AM, Sciallis AP (2020). Molecular characterization of uterine and ovarian tumors with mixed epithelial and germ cell features confirms frequent somatic derivation. Mod Pathol.

[14] Hodroj K, Stevovic A, Attignon V (2021). Molecular characterization of ovarian yolk sac tumor (OYST). Cancers (Basel).

[15] Numan TA, Ronnett BM, Haley L (2025). Clinicopathologic and molecular analysis of malignant neoplasms with yolk sac tumor differentiation in women 40 years of age and older. Am J Surg Pathol.

